# Significance of serum protein electrophoresis in the diagnosis of Tsukayama type IV periprosthetic joint infection

**DOI:** 10.3389/fcimb.2024.1343338

**Published:** 2024-04-30

**Authors:** Xinzhe Lu, Mingwei Hu, Hao Xu, Haining Zhang, Shuai Xiang

**Affiliations:** Department of Joint Surgery, The Affiliated Hospital of Qingdao University, Qingdao, Shandong, China

**Keywords:** periprosthetic joint infection, serum protein electrophoresis, diagnosis, α1 globulin, Tsukayama type IV

## Abstract

**Objectives:**

To investigate the efficacy of serum protein electrophoresis (SPE) in the diagnosis of periprosthetic joint infection (PJI) after hip and knee arthroplasty.

**Methods:**

The medical records of patients undergoing hip and knee arthroplasty at a class A tertiary hospital between August 2013 and January 2021 were retrospectively investigated. A total of 179 patients were included and divided into two groups: 66 patients in the PJI group and 113 patients in the aseptic loosening (AL) group. Serum C-reactive protein (CRP), erythrocyte sedimentation rate (ESR), D-dimer, Fibrinogen, Serum albumin and the proportion of serum protein in SPE were compared between the two groups. The diagnostic sensitivity and specificity were determined using the receiver operating characteristic (ROC) curve, and the diagnostic value was compared using the area under the ROC curve (AUC).

**Results:**

There was no significant difference in age, sex and body mass index (BMI) between PJI group and AL group (P>0.05), but there was significant difference in the ratio of hip to knee (X^2 = ^22.043, P<0.001). The CRP, ESR, D-dimer, Fibrinogen and the proportion of α1 globulin band in PJI group was 22.99(10.55,40.58) mg/L, 37.00(23.00,61.70) mm/h, 790.00(500.00,1500.00) ng/ml, 4.84(3.81,5.55) g/L and 5.80(5.00,7.73) % which was higher than that in AL group [1.89(0.50,4.12) mg/L, U=7.984, P<0.001; 10.10(7.00,16.90) mm/h, U=8.095, P<0.001; 570.00(372.50,780.00) ng/ml, U=3.448, P<0.001; 2.84(2.45,3.43) g/L, U=8.053, P<0.001 and 4.20(3.90,4.80) %, U=8.154, P<0.001]. The Serum albumin and the proportion of Albumin band in PJI group was 36.10(33.10,39.00) g/L and 49.00(44.95,52.20) % which was lower than that in AL group [38.10(34.00,41.10) g/L, U=-2.383, P=0.017 and 54.40(51.55,56.70) %, U=-6.162, P<0.001]. The proportion of In PJI group, the AUC of proportion of α1 globulin was 0.8654, which was equivalent to CRP (0.8698), ESR (0.8680) and outperformed that of fibrinogen (0.8025).

**Conclusions:**

Elevated proportion of α1 globulin in SPE presented with good diagnostic value for Tsukayama type IV PJI, and its accuracy was comparable to those of ESR and CRP. And α1 globulin can assist with CRP and ESR to determining the timing of second-stage revision.

## Introduction

Knee and hip arthroplasty is the most effective treatment for advanced joint diseases, aiming to relieve pain and restore joint function (Ethgen et al., 2004). However, periprosthetic joint infection (PJI) is one of the major reasons leading to revision procedures after hip and knee arthroplasties (Bozic et al., 2010; Kelmer et al., 2021), which are associated with enormous trauma, higher medical expense, and increased mortality (Kurtz et al., 2008; Bozic et al., 2010; Kelmer et al., 2021). Therefore, diagnosis of PJI is crucial to reduce the damage. The modified musculoskeletal infection society (MSIS) criteria in 2018 is now the most widely accepted standard for diagnosing PJI (Guan et al., 2019). Notably, serum D-dimer was newly introduced into this modified criterion and the new scoring system improved the diagnostic sensitivity from 79.3% to 94.9%, with a specificity of 95.2%. Although the significance of D-dimer for diagnosing PJI has been increasingly questioned in the past few years, the exploration for an accurate, less-invasive serum marker is still needed.

Recently, serum fibrinogen has been regarded as a promising marker for diagnosing PJI, with comparable accuracy to that of CRP and ESR (Huang et al., 2021). However, as a coagulative marker, the diagnostic value of fibrinogen may be impaired by coagulation disorders, particularly thrombosis after knee or hip arthroplasty (de Moerloose et al., 2010). Tsukayama type I is positive intraoperative cultures: when at least two specimens that had been obtained at the time of the revision operation were positive on culture; Tsukayama type II is early postoperative infection: a wound infection that developed less than one month after the operation; Tsukayama type III is acute hematogenous infection: which associated with a documented or suspected antecedent bacteremia and was characterized by an acute onset of symptoms in the affected joint with the prosthesis; Tsukayama type IV is late chronic infection: one that developed one month or more after the index operation and that had an insidious clinical course (Tsukayama et al., 1996). Unlike the acute and short-term outburst of Tsukayama type II and type III PJI, the progression of Tsukayama type IV PJI is usually chronic and the gradual loss of joint mobility might lead to higher incidence of thrombosis (Yu et al., 2021; Rowland et al., 2023). As a result, the diagnostic value of coagulative factors for Tsukayama type IV PJI is further undermined and new less-invasive markers are needed.

Given the wasting condition of infectious diseases, serum proteins are increasingly focused during the diagnosis and treatment of multiple infectious and inflammatory diseases (Chen et al., 2020a). Several recent studies, including our study, have reported serum protein as potential markers for diagnosing PJI (Wang et al., 2021; Choe et al., 2022; Shang et al., 2022). Although the independent diagnostic value of overall serum globulin was only fair in our previous study, the potential of serum globulin for diagnosing PJI is still attractive since globulin is related to immune reaction and has little correlation with coagulative system. Generally, there are at least four types of serum globulin, and serum protein electrophoresis (SPE) is required to distinguish them. Previous studies have demonstrated that the changed proportion of different proteins in SPE was correlated with infection or inflammatory conditions (O'Connell et al., 2005). However, the usage of SPE for diagnosing PJI has never been reported.

Herein, this study aims to assess the diagnostic value of SPE in PJI and compare their accuracy with that of conventional inflammatory markers (CRP, ESR) and coagulation factors (D-dimer, fibrinogen).

## Materials and methods

**Figure 1:**
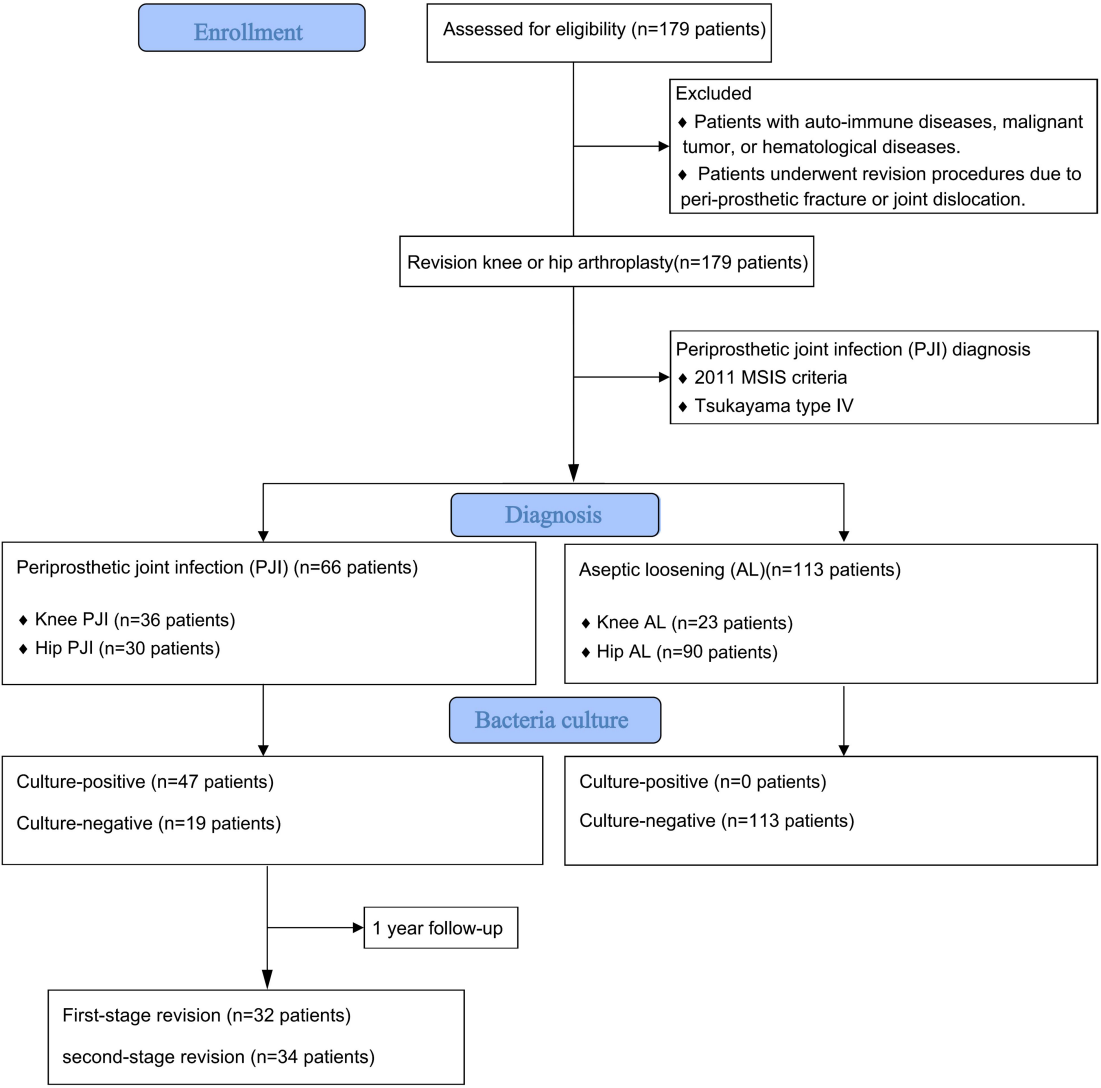
follow chart of retrospective study.

This study was approved by the institutional review board. The medical records of patients who underwent single-stage or two-stage revision knee or hip arthroplasty between August, 2013 and January, 2021 were retrospectively reviewed. Inclusion criteria: ① Patients underwent a first stage implant removal due to PJI (Tsukayama type IV) based on the 2011 MSIS criteria:(1) there is a sinus tract communicating with the prosthesis; or (2) a pathogen is isolated by culture from 2 or more separate tissue or fluid samples obtained from the affected prosthetic joint; or (3) when 4 of the following 6 criteria exist: (a) elevated serum erythrocyte sedimentation rate and serum C-reactive protein (CRP) concentration, (b) elevated synovial white blood cell count, (c) elevated synovial polymorphonuclear percentage (PMN%), (d) presence of purulence in the affected joint, (e) isolation of a microorganism in one culture of periprosthetic tissue or fluid, or (f) greater than 5 neutrophils per high-power field in 5 high-power fields observed from histologic analysis of periprosthetic tissue at ×400 magnification (Workgroup Convened by the Musculoskeletal Infection S, 2011); ② Patients underwent revision procedure due to aseptic loosening (AL). Exclusion criteria: ① Patients with auto-immune diseases, malignant tumor, or hematological diseases. ② Patients underwent revision procedures due to peri-prosthetic fracture or joint dislocation (**Figure 1**).

A total of 179 patients were included in this study. This study has been approved by the Ethics Committee of hospital (QYFFWZLL26266). 66 cases (36.9%) were classified as PJI group and 113 cases (63.1%) were classified as AL group. Demographic data of the included patients is listed in **Table 1**. Fasting blood samples, as well as intra-articular aspiration of all patients were collected on the day of admission and sent to clinical laboratory for routine preoperative screening and pathogen culture. The conventional inflammatory markers (CRP and ESR), coagulative markers (D-Dimer and fibrinogen), the results of SPE and the bacterial and fungal culture results were recorded for further analysis.

**Table 1:** Demographic information of the included patients (n=179).

Group	Gender		Age (year)	BMI (kg/m^2^)	Joint	
	Male	Female			Knee	Hip
PJI (n=66)	33	33	67.08 ± 10.61	25.74 ± 4.94	36	30
AL (n=113)	61	52	69.89 ± 9.92	25.28 ± 3.64	23	90
Statistical value	0.265^a^		-1.857^b^	0.711^b^	22.043^a^	
P value	0.607		0.065	0.478	<0.001	

PJI, periprosthetic joint infection; BMI, body mass index.

a X^2^ value.

b t value.

CRP was determined by ARIST0 specific protein analyzer (Goldsite, Inc., Shenzhen, China) and BK051 whole-process C-reactive protein determination kit (Goldsite, Inc., Shenzhen, China), and analyzed by supporting software. The reference range of CRP normal value is < 5mg/L.

ESR was determined by Monitor-100 automatic erythrocyte sedimentation rate analyzer (VITAL Diagnostics S.r.l, Inc., Forli, Italy) and analyzed by supporting software. The reference range of ESR normal value is 0-15mm/h for male and 0-20mm/h for female.

D-dimer was determined by sysmex CS-5100 automatic hemagglutination analyzer (sysmex, Inc., Kobe, Japan) and D-dimer determination kit (latex immunoturbidimetry) (SUNBIO, Inc., Shanghai, China), and analyzed by supporting software. The reference range of D-Dimer normal value is: < 5.5mg/LFibrinogen was determined by STA-R MAX automatic hemagglutination analyzer (Stago, Inc., Paris, France) and FIB kit (Stago, Inc., Paris, France), and analyzed by supporting software. the reference range of Fibrinogen normal value is 2-4g/L.

SPE was determined by Spice TOUCH automatic electrophoresis analyzer (Helena Laboratories, Inc., Texas, USA) and REP/SPIFE SP 300 kit (Helena Laboratories, Inc., Texas, USA), and analyzed by supporting software. The reference range of SPE normal value is: albumin 54.1-66.7%, α 1 globulin 1.4-5.0%, α 2 globulin 6.5-12.6%, β globulin 9.0-16.2%, gamma globulin 10.1-22.9%.

### Statistical analysis

All statistical analyses were performed using SPSS version 26.0 (IBM Corp., Armonk, NY). For continuous variables, after the normality test, the variables obeying normal distribution (including age and BMI) were expressed in the form of means ± standard deviations (SD), and the independent sample t-test was used for comparison between PJI group and AL group. Variables that do not obey normal distribution (including CRP, ESR, D-dimer, fibrinogen, serum albumin, serum globulin and albumin band, α1 globulin band, α2 globulin band, β globulin band and γ globulin band is expressed by M (Q1, Q3), Mann-Whitney U test was used for comparison between the two groups. For categorical variables (including gender, joint type) is expressed by the number of cases, and the comparison between groups is made by X^2^ test. The statistical significance threshold was set at P<0.05. The biomarkers’ areas under the curves (AUCs), 95 percent confidence interval (CI), and sensitivity and specificity were calculated using receiver operating characteristic (ROC) curves. The optimal biomarker cutoff values were then calculated using the Youden index. GraphPad Prism (version9.0; GraphPad Software Inc., San Diego, CA) was utilized to create the diagrams.

## Results

There was no significant difference between the PJI and AL groups in terms of gender, age, or BMI, but there was a significant difference in joint type (P<0.05, **Table 1**). The liver function of all patients was determined as Child-Pugh A5 or A6.

### Comparison between PJI group and AL group

**Figure 2:**
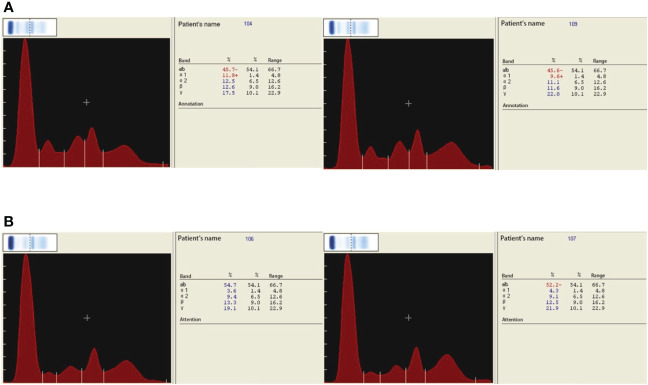
**(A)** SPE of PJI group. **(B)** SPE of AL group. Symbol + means higher than the normal value, the normal value has been shown in the section Materials and Methods.

**Figure 3:**
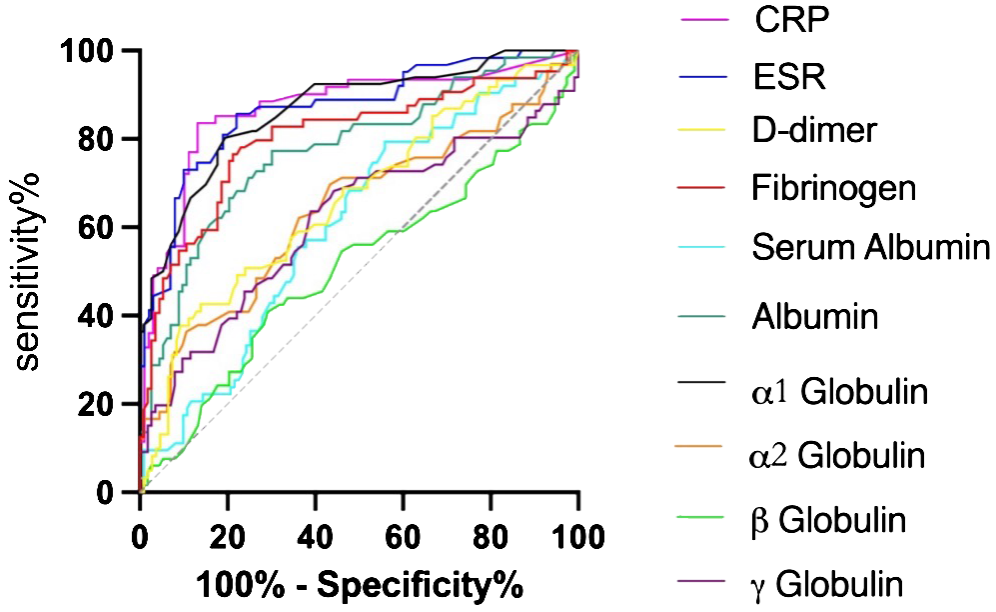
ROC curve of CRP, ESR, D-dimer, Fibrinogen, serum protein and SPE.

In accordance to the previous studies, serum CRP, ESR, D-dimer, and fibrinogen of PJI group were significantly higher, with a diagnostic AUC of 0.8698, 0.8680, 0.6617, and 0.8025, respectively. Correspondingly, the proportion of α1 globulin, α2 globulin, and *γ* globulin in SPE of PJI group were also significantly higher (**Figure 2**). Notably, the diagnostic AUC of α1 globulin reached 0.8654, which was comparable to that of CRP and ESR, and outperformed fibrinogen (**Table 2**). The overall serum albumin and the proportion of albumin in SPE of PJI group were both significantly lower than those of AL group, with a diagnostic AUC of 0.6088 and 0.7763, respectively. Our results also demonstrated that the proportion of β globulin in SPE has little correlation with PJI, while the α2 globulin and γ globulin only displayed poor diagnostic value for PJI (**Table 2**). The optimal cutoff value, sensitivity and specificity of the target markers are shown in **Table 3** and the ROC curves are shown in **Figure 3**. Evidently, the sensitivity and specificity of the α1 globulin in SPE were equivalent to those of CRP and ESR and surpassed those of fibrinogen. The combinational diagnostic results are listed in **Table 4**. When combining the proportion of α1 globulin with CRP, ESR and the proportion of albumin in SPE, the diagnostic value all reached “excellent”, with an AUC more than 0.9. We further analyzed the diagnostic value of the markers for PJI of hip or knee joint independently. And the power analysis of α1 globulin in hip group was 0.81. Although the AUC of α1 globulin for diagnosing knee PJI was slightly lower than that of CRP and ESR (**Table 5**), it performed better than CRP and ESR when diagnosing hip PJI (**Table 6**).

**Table 2:** Comparison of serum parameters and SPE between PJI group and AL group.

Markers	PJI (n=66)	AL (n=113)	U value	P value
CRP (mg/L)	22.99 (10.55,40.58)	1.89 (0.50,4.12)	7.984	<0.001
ESR (mm/h)	37.00 (23.00,61.70)	10.10 (7.00,16.90)	8.095	<0.001
D-dimer (ng/mL)	790.00 (500.00,1500.00)	570.00 (372.50,780.00)	3.448	<0.001
Fibrinogen (g/L)	4.84 (3.81,5.55)	2.84 (2.45,3.43)	8.053	<0.001
Serum albumin (g/L)	36.10 (33.10,39.00)	38.10 (34.00,41.10)	-2.383	0.017
Albumin (%)	49.00 (44.95,52.20)	54.40 (51.55,56.70)	-6.162	<0.001
α1 Globulin (%)	5.80 (5.00,7.73)	4.20 (3.90,4.80)	8.154	<0.001
α2 Globulin (%)	10.80 (9.43,12.80)	9.80 (8.85,11.25)	3.150	0.002
*β* Globulin (%)	12.30 (11.10,13.75)	12.10 (11.30,13.30)	0.202	0.840
*γ* Globulin (%)	20.80 (17.58,24.43)	18.90 (17.05,21.30)	2.559	0.010

ESR, erythrocyte sedimentation rate; CRP, C-reactive protein.

**Table 3:** The diagnostic value of serum markers for PJI.

Markers	AUC (95% CI)	Optimal cutoff value	Sensitivity (%)	Specificity (%)
CRP (mg/L)	0.8698 (0.8065-0.9330)	8.545 mg/L	83.61	86.87
ESR (mm/h)	0.8680 (0.8093-0.9267)	17.6 mm/h	85.71	78.00
D-dimer (ng/mL)	0.6617 (0.5746-0.7488)	970.0 ng/mL	42.62	86.11
Fibrinogen (g/L)	0.8025 (0.7286-0.8764)	3.665 g/L	76.56	78.76
Serum albumin (g/L)	0.6088 (0.5288-0.6949)	39.45 g/L	79.37	44.14
Albumin (%)	0.7763 (0.7033-0.8493)	52.25%	77.27	69.91
α1 Globulin (%)	0.8654 (0.8087-0.9222)	4.85%	80.30	80.53
α2 Globulin (%)	0.6413 (0.5528-0.7297)	10.05%	69.70	56.64
*β* Globulin (%)	0.5091 (0.4174-0.6007)	12.95%	40.91	70.80
*γ* Globulin (%)	0.6148 (0.5233-0.7062)	19.9%	63.64	61.06

PJI, periprosthetic joint infection; ESR, erythrocyte sedimentation rate; CRP, C-reactive protein.

**Table 4:** Combinational diagnostic value of α1 globulin with CRP, ESR and albumin in SPE.

	AUC (95% CI)	Sensitivity (%)	Specificity (%)
α1 Globulin and CRP	0.915 (0.865-0.965)	83.6	90.0
α1 Globulin and ESR	0.938 (0.900-0.976)	93.7	84.0
α1 Globulin and albumin in SEP	0.901 (0.854-0.948)	89.4	78.8

PJI, periprosthetic joint infection; ESR, erythrocyte sedimentation rate; CRP, C-reactive protein; SPE, serum protein electrophoresis; CI, confidence interval.

**Table 5:** Diagnostic value of the serum markers for knee PJI.

Markers	PJI (n=36)	AL (n=23)	P value	AUC (95% CI)	Sensitivity (%)	Specificity (%)
CRP (mg/L)	17.43 (8.66,30.80)	2.24 (0.54,4.13)	<0.001	0.8610 (0.7572-0.9648)	79.41	95.45
ESR (mm/h)	35.75 (19.08,58.85)	9.98 (6.75,14.20)	<0.001	0.8832 (0.7967-0.9698)	83.33	90.91
D-dimer (ng/mL)	1120.00 (720.00,1662.50)	525.00 (317.50,792.50)	<0.010	0.7534 (0.6171-0.8897)	78.79	72.73
Fibrinogen (g/L)	4.37 (3.38,5.34)	2.91 (2.45,3.47)	<0.001	0.8248 (0.7152-0.9345)	74.29	91.30
Serum albumin (g/L)	35.65 (31.10,37.85)	38.70 (32.60,41.10)	0.048	0.6262 (0.4776-0.7748)	63.89	65.22
Albumin (%)	49.00 (44.50,52.83)	53.20 (50.80,56.20)	0.002	0.7446 (0.6178-0.8713)	72.22	73.91
α1 Globulin (%)	5.60 (4.68,7.68)	4.30 (3.90,4.50)	<0.001	0.8466 (0.7458-0.9475)	75.00	91.30
α2 Globulin (%)	10.60 (8.68,12.38)	9.90 (9.40,10.80)	0.280	0.5839 (0.4370-0.7308)	58.33	69.57
*β* Globulin (%)	11.45 (10.33,13.28)	12.50 (11.50,13.80)	0.079	0.6365 (0.4906-0.7823)	66.11	73.91
*γ* Globulin (%)	20.90 (19.33,25.33)	18.20 (17.00,21.50)	0.025	0.6745 (0.5331-0.8159)	77.78	60.87

PJI, periprosthetic joint infection; ESR, erythrocyte sedimentation rate; CRP, C-reactive protein.

**Table 6:** Diagnostic value of the serum markers for hip PJI.

Markers	PJI (n=30)	AL (n=90)	P value	AUC (95% CI)	Sensitivity (%)	Specificity (%)
CRP (mg/L)	26.69 (12.60,48.97)	1.59 (0.05,4.18)	<0.001	0.8981 (0.8345-0.9618)	85.19	83.33
ESR (mm/h)	39.00 (28.00,63.70)	10.50 (6.88,19.00)	<0.001	0.8599 (0.7741-0.9457)	74.07	87.18
D-dimer (ng/mL)	600.00 (430.00,1110.00)	585.00 (387.50,785.00)	0.558	0.5365 (0.4146-0.6584)	24.14	93.02
Fibrinogen (g/L)	4.89 (4.10,5.87)	2.83 (2.44,3.42)	<0.001	0.7998 (0.6885-0.9111)	82.76	76.67
Serum albumin (g/L)	37.40 (33.45,39.60)	38.10 (34.20,41.10)	0.362	0.5566 (0.4372-0.6761)	75.86	45.45
Albumin (%)	49.20 (45.38,52.20)	54.55 (51.58,56.95)	<0.001	0.7904 (0.6905-0.8902)	70.00	81.11
α1 Globulin (%)	6.15 (5.10,8.13)	4.20 (3.90,4.80)	<0.001	0.9031 (0.8452-0.9611)	86.67	77.78
α2 Globulin (%)	11.15 (9.98,12.88)	9.70 (8.70,11.30)	0.002	0.6898 (0.5726-0.8071)	13.33	100.00
*β* Globulin (%)	13.05 (12.23,13.09)	12.00 (11.28,13.10)	0.009	0.6585 (0.5489-0.7681)	76.67	57.78
*γ* Globulin (%)	20.60 (15.20,24.25)	19.15 (17.18,21.15)	0.413	0.5000 (0.4154-0.5846)	95.56	4.44

PJI, periprosthetic joint infection; ESR, erythrocyte sedimentation rate; CRP, C-reactive protein.

### Comparison between culture-positive and culture-negative

Among the 66 patients diagnosed with PJI, the culture results were recorded and 47 patients were culture-positive (71.21%) and 19 patients were culture-negative. No significant difference in CRP, ESR, fibrinogen, the proportion of albumin, α1 globulin, α2 globulin, β globulin and γ globulin in SPE between culture-positive and culture-negative groups. However, the D-dimer in SPE of culture-positive patients were significantly higher than those of culture-negative patients, while the serum albumin was significantly lower (**Table 7**). Moreover, 34 patients have been followed up for at least one year after completing a two-stage revision procedure including a first-stage implant removal, thorough debriment, antibiotics treatment followed by a second-stage revision and no reinfection was observed. The serum levels of CRP, ESR, fibrinogen and the proportion of α1 globulin in SPE before the second-stage revision were significantly decreased than those before the first-stage implant removal, indicating their potential use in prediction the timing of the second-stage revision (**Table 8**).

**Table 7:** Comparison of the serum markers between culture-positive and culture negative PJI.

Markers	Culture-positive (n=47)	Culture-negative (n=19)	U value	P value
CRP (mg/L)	25.73 (11.14,47.45)	13.24 (25.77,8.95)	1.580	0.114
ESR (mm/h)	41.05 (24.88,65.78)	34.10 (17.25,42.50)	1.643	0.100
D-dimer (ng/mL)	910.00 (590.00,1650.00)	605.00 (342.50,1167.50)	2.159	0.031
Fibrinogen (g/L)	5.01 (3.83,5.70)	3.90 (3.68,4.98)	1.747	0.081
Serum albumin (g/L)	35.00 (31.10,37.75)	39.10 (35.45,43.50)	-2.852	0.004
Albumin (%)	49.00 (44.40,52.00)	50.00 (48.20,54.10)	-1.452	0.147
α1 Globulin (%)	5.60 (4.90,7.60)	6.00 (4.60,7.80)	-0.333	0.739
α2 Globulin (%)	10.70 (9.50,12.40)	11.70 (8.30,14.70)	-0.467	0.640
*β* Globulin (%)	12.30 (10.80,13.40)	13.10 (11.20,14.10)	-1.204	0.229
*γ* Globulin (%)	21.10 (19.20,25.60)	20.00 (17.30,22.60)	1.877	0.061

PJI, periprosthetic joint infection; ESR, erythrocyte sedimentation rate; CRP, C-reactive protein.

**Table 8:** Serum markers in patients with successfully controlled PJI (n=34).

Markers	First-stage implant removal	Second-stage revision	U value	P value
CRP (mg/L)	16.37 (8.82,28.85)	1.68 (1.02,3.19)	4.069	<0.001
ESR (mm/h)	34.20 (22.30,62.00)	10.00 (7.00,17.00)	5.332	<0.001
D-dimer (ng/mL)	945.00 (560.00,1440.00)	810.00 (395.00,1387.50)	0.894	0.372
Fibrinogen (g/L)	4.39 (3.76,5.37)	2.63 (2.26,3.27)	5.498	<0.001
Serum albumin (g/L)	35.75 (33.68,38.13)	38.50 (35.60,40.80)	-1.780	0.075
Albumin (%)	50.40 (47.7,54.10)	52.30 (50.10,55.10)	-1.375	0.169
α1 Globulin (%)	5.60 (4.90,7.70)	4.40 (3.80,4.90)	3.817	<0.001
α2 Globulin (%)	10.80 (9.50,12.30)	9.70 (8.60,11.30)	1.575	0.115
*β* Globulin (%)	12.30 (10.80,13.30)	13.10 (12.00,13.80)	-1.869	0.062
*γ* Globulin (%)	20.20 (17.80,24.40)	19.50 (18.10,22.50)	0.337	0.736

PJI, periprosthetic joint infection; ESR, erythrocyte sedimentation rate; CRP, C-reactive protein. Wilcoxon’s signed-rank test for paired samples.

## Discussion

This study investigated the diagnostic value of CRP, ESR, D-dimer, fibrinogen and the proportion of serum proteins in SPE for Tsukayama type IV PJI. Our results demonstrated that the diagnostic value of the proportion of α1 globulin in SPE for Tsukayama type IV PJI was equivalent to that of ESR and CRP, and surpassed the diagnostic value of fibrinogen, a promising diagnostic marker reported recently. The proportion of α1 globulin was also assistant in determining the timing of second-stage revision. For the first time, the application of SPE was introduced in the diagnosis of Tsukayama type IV PJI by our study.

The effort of exploring a determining, less-invasive diagnostic marker for PJI has never stopped. Since the involvement of serum D-dimer into the modified MSIS criteria in 2018, serum markers, especially coagulative markers, other than traditional inflammatory markers has been extensively investigated (Hu et al., 2020). Recent studies have recommended serum fibrinogen as an alternative for D-dimer since the diagnostic value of D-dimer was inferior to that of fibrinogen (Pan et al., 2021). However, one of the innate flaws of the coagulative markers is that their diagnostic values might be deteriorated by some coagulative disorders such as venous thrombosis or the application of anti-coagulants (Chen et al., 2020b). The good to excellent diagnostic value of fibrinogen are established on excluding patients who have abnormal coagulative conditions (Chen et al., 2020b). However, the long-term consistent pain caused by Tsukayama type IV PJI or aseptic loosening limited the joint mobility and might increase the incidence of venous thrombosis. Thus, in this study, we included all patients who were diagnosed with Tsukayama type IV PJI regardless whether there were factors influencing the coagulative system. Our results demonstrated that the AUC of fibrinogen and D-dimer was 0.8025 and 0.6617, respectively. Obviously, the involvement of patients might have thrombus affected the diagnostic accuracy of fibrinogen and D-dimer. As a result, markers ignore the influence of thrombus might be more suitable for distinguishing Tsukayama type IV PJI from aseptic loosening.

The application of serum proteins, albumin and globulin, for diagnosing PJI have been increasingly discussed recently. Considering the tight relationship between globulin and infection, we suppose that the globulin might be a promising marker to diagnose PJI. However, there are at least four types of globulins in human serum, and SPE is used to distinguish them. SPE is a routine, convenient clinical test and has been widely utilized for diagnosing liver diseases (Papadopoulos et al., 1990), renal diseases (Chew et al., 1999), multiple myeloma (Dejoie et al., 2016), and connective tissue disorders (Madda et al., 2019). And recent studies also corelated the alteration of SPE with infection (Davido et al., 2016). Our previous research has also reported a good PJI diagnostic value of albumin to globulin ratio and a fair diagnostic value of overall serum globulin independently (Shang et al., 2022). However, the application of SPE for diagnosing PJI has never been reported. The electrophoresis method used in this study was agarose gel (Dennis et al., 1987), and five protein bands were isolated, including albumin, α1 globulin, α2 globulin, β globulin and γ globulin.

Serum albumin maintains the nutritional status and the osmotic pressure of plasma. Guo et al. noted that hypoproteinemia is an independent risk factor for PJI (Guo et al., 2020). Bohl et al. also revealed that patients with hypoalbuminemia had a higher incidence of PJI in the early stage after revision (Bohl et al., 2016). However, the diagnostic value of serum albumin independently is still controversial. Although Hyonmin et al. pointed out that serum albumin has high diagnostic value in mild PJI (Choe et al., 2022), most recent studies failed to confirm its independent diagnostic value. As a result, serum albumin could only be used as a numerator or denominator in a calculated ratio such as albumin to globulin ratio (Shi et al., 2021; Shang et al., 2022). However, it is inconvenient to calculate a ratio during clinical practice. This study also revealed that the absolute amount of serum albumin and the proportion of albumin in SPE of PJI group were both decreased. However, the proportion of albumin in SPE presented with good diagnostic value, with an AUC of 0.7763 (sensitivity 77.27%, specificity 69.91%), while the diagnostic value of absolute amount of serum albumin was poor (AUC 0.6088, sensitivity 79.37%, specificity 44.14%). Although this result seems controversial, it is easy to interpret. Most patient with aseptic loosening usually suffers a relative long-term joint pain, which was also wasting and could be reflected by decreased absolute amount of albumin. And the wasting condition of aseptic loosening is obviously slighter than PJI, since the serum albumin was still significantly higher. Meanwhile, the infectious condition of the patients with PJI also changes the composition of the serum protein, which is reflected by the significant alteration of the proportion of albumin in SPE, leading to a better diagnostic value than that of absolute mount of serum albumin.

α1 globulin is mainly composed of α1 acid glycoprotein and α1 antitrypsin. α1 acid glycoprotein is an acute phase protein produced by liver and peripheral tissue in response to systemic inflammation. The serum concentration of α1 acid glycoprotein rises in response to systemic tissue injury, inflammation, or infection, and it modulates the immune system (Ceciliani and Lecchi, 2019; Fournier et al., 2000). α1 antitrypsin is also reported to be a versatile factor with anti-inflammatory, immunomodulatory and anti-infectious function (de Serres and Blanco, 2014). Both α1 acid glycoprotein and α1 antitrypsin have been reported to elevate during bacterial, fungal and viral infection (Naik et al., 2020; Wettstein et al., 2021). However, these two proteins are not routinely tested and there is no study focusing on their diagnostic value for PJI. Although the electrophoresis used in this study unable to distinguish these two proteins, we found that α1 globulin level of the PJI group was significantly higher than that of the AL group, with a diagnostic AUC of 0.8654 (sensitivity 80.3%, specificity 80.5%), which was comparable to that of CRP and ESR. In particular, we found the diagnostic value of α1 globulin in SPE outperformed the ESR and CRP, further confirming the promising role of SPE used for PJI diagnosing. The combinational AUCs of α1 globulin with CRP, ESR and albumin in SPE all reached 0.9, indicating excellent combinational diagnostic values with high sensitivity and specificity.

Although single-stage revision for PJI is increasingly accepted worldwide, the second-stage revision regimen including first-stage implant removal, debriment, antibiotics treatment followed by a second-stage revision is still the gold standard for Tsukayama type IV PJI (Tsukayama et al., 2003). The result of pathogen culture determines the intra-operative during first-stage implant removal, as well as postoperative antibiotics selection. However, the incidence of culture-negative PJI has been reported to be 5% to 42% (Palan et al., 2019). Antibiotics treatment prior to sample collection and low-toxicity infection are major reasons contribute to negative culture results. On occasions, the first-stage procedure might be performed in absence of culture results since up to 16 days are needed to detect certain low-toxic pathogens (Barry et al., 2019). In our study, we found that there was no significant difference in CRP, ESR, fibrinogen and proportion of α1 globulin band between culture positive group and culture negative group. The result showed that the proportion of α1 globulin band in SPE was not affected by the culture result. It can also be used together with CRP, ESR and fibrinogen to distinguish AL from culture negative PJI as early as possible.

The timing of second-stage revision is also important to prevent recurrent infection and the lack of a determining marker for predicting the timing is still bothering. ESR and CRP are the most widely markers to determine the timing. Ghanem et al. found that the inflammatory markers of the PJI patients who were successfully treated demonstrated a decreasing trend (Ghanem et al., 2009). Ryu et al. also found that PJI patients with high serum inflammatory markers also had a higher recurrence risk (13.9%: 4%) (Ryu et al., 2014). However, Jeffrey et al. noted that the timing of revision surgery should be evaluated comprehensively rather than rely on the levels of ESR and CRP (Stambough et al., 2019). In this study, we found that in those patients with successfully controlled PJI, the decreasing trend of the proportion of α1 globulin in SPE was similar to those of ESR and CRP. The proportion of α1 globulin in SPE was significantly lower after first-stage implants removal and successful antibiotic treatment. Our results demonstrated that α1 globulin in SPE can be used as a referential marker for evaluating the infection control and deciding the timing second-stage revision.

There are still some limitations in this study. First of all, the serum proteins are mainly produced by the liver, and the SPE results might be affected in patients with severe liver injury. In this study, all patients included were qualified for revision procedure and the preoperative liver function was determined as Child-Pugh A5 or A6. As a result, further researches are needed to investigate the effectiveness of SPE in patients with PJI and a concurrent Child-Pugh B or C score. Moreover, the agarose gel electrophoresis technique used in this study can only distinguish five types of serum proteins. Although we have demonstrated the significance of α1 globulin for PJI diagnosis, further studies are needed to clarify the value of specific protein separately.

In conclusion, our results provided the first report on the effectiveness of SPE for diagnosing Tsukayama type IV PJI, predicting negative culture results, and determining the timing of second-stage revision. α1 globulin in SPE is a direct, convenient and less-invasive marker for diagnosing PJI, can be extracted together with other serological indicators without the need for joint cavity puncture, with an equivalent diagnostic value to that of CRP and ESR.

## Data availability statement

The original contributions presented in the study are included in the article/supplementary materials. Further inquiries can be directed to the corresponding author.

## Ethics statement

The studies involving humans were approved by the Ethics Committee of the Affiliated Hospital of Qingdao University (QYFFWZLL26266). The studies were conducted in accordance with the local legislation and institutional requirements. The participants provided their written informed consent to participate in this study.

## Author contributions

XL: Writing – original draft. MH: Writing – original draft. HX: Writing – review & editing. HZ: Writing – review & editing. SX: Writing – review & editing.
